# School refusal profiles maintained by negative reinforcement and their relationship with self-perceived health

**DOI:** 10.3389/fpsyg.2024.1340010

**Published:** 2024-03-18

**Authors:** Javier Martínez-Torres, Carolina Gonzálvez, Nuria Antón

**Affiliations:** Department of Developmental Psychology and Didactics, University of Alicante, San Vicente del Raspeig, Spain

**Keywords:** school refusal, health, self-perceived health, latent profiles, school attendance, negative reinforcement

## Abstract

Health alterations and school refusal behavior may significantly affect student evolution in all areas of student lives. The objective of this study was to use latent profile analysis to identify school refusal profiles sustained by negative reinforcement and to determine their relationship with distinct self-perceived health variables (Satisfaction, Well-being, Resilience, Performance, and Risk-Taking). The School Refusal Assessment Scale-Revised (SRAS-R) and the Child Health and Illness Profile (CHIP-CE/CRF) were administered to 737 students (60.9% male) aged between 8 and 10 (*M* = 8.76, *SD* = 0.74). Three profiles of school refusal maintained by negative reinforcement were obtained: no risk, moderate risk, and high risk. It was confirmed that school refusal through negative reinforcement correlates negatively with health dimensions, also finding that a higher risk profile for school refusal is associated with lower levels of self-perceived health. Similarly, it was determined that the high-risk profile is the most maladaptive, with significantly lower data in four of the five self-perceived health dimensions that were evaluated. In conclusion, remaining in situations with no or moderate risk of school refusal due to negative reinforcement encourages higher levels of self-perceived health, while being at high risk of school refusal due to negative reinforcement is associated with worse self-perceived health.

## 1 Introduction

School Attendance Problems (SAPs) is a generic term used to refer to a series of problematic school absences (Adams et al., 2022). Efforts have been made to reach a consensus on the different concepts used, with authors such as Heyne et al. (2019) and Gren Landell (2021) identifying various topics that have been more widely accepted in the scientific community: S*chool refusal*, absence caused by various types of emotional problems; *Truancy*, unauthorized lack of attendance with no excuse offered; S*chool withdrawal*, that which is the result of decision by parents or guardians; and *School exclusion*, absenteeism stemming from the school itself.

Evidence reveals that all types of absenteeism, including that resulting from school refusal, are negatively associated with academic performance (Klein et al., 2022). Furthermore, beyond school progress, it has been confirmed that school attendance problems during the first 10 years of a student’s education may lead to numerous long-term negative effects in other areas, such as economic and health areas (Ansari et al., 2020).

### 1.1 School refusal profiles

Kearney and Silverman (1993) created a functional model of school refusal behavior. The authors proposed that four functional principles of school refusal behavior exist, related to the type of reinforcement maintaining them, sustained by either positive reinforcement (which increases the behavior), or negative reinforcement (the removal of which increases the frequency of the response). According to this model, the first two factors are maintained by negative reinforcement, with factor I referring to student refusal that is marked by avoiding or evading school situations due to a negative affectivity toward the school and factor II being marked by anxiety caused by evaluations and/or social aversion. The factors maintained by positive reinforcement correspond to the search for attention from third parties (factor III), and to obtaining tangible rewards or privileges outside the institution (factor IV).

To evaluate these four functional conditions, the School Refusal Assessment Scale was designed (SRAS; Kearney and Silverman, 1993), whose revised version, the School Refusal Assessment Scale-Revised (SRAS-R; Kearney, 2002) has become one of the most widely recognized scales used to assess scholastic attendance issues in general, and specifically, school refusal (Gallé-Tessonneau, 2021).

Diverse profiles of students with SAPs have been found, such as those evidencing a high risk of school refusal (De Aldaz et al., 1987), those investigating behavioral and diagnostic (Fernando and Perera, 2012), psychopathological (Romani et al., 2017), clinical (Nayak et al., 2018), and behavioral (Nwosu et al., 2022) profiles. There has also been a growing interest in the identification of different school refusal (SR) profiles. Fernández-Sogorb et al. (2023) identified diverse SR profiles in youth aged 12 to 18, when carrying out a review of eight studies, all using a Spanish population (except for two carried out in Latin America). Thus, they report that three profiles have been commonly detected, the so-called “non-school refusal or low school refusal,” characterized by low scores on various factors of the SRAS-R; a “mixed or anxious school refusal profile,” corresponding to high or moderately high scores on the first three dimensions of the SRAS-R; and “high school refusal” and “moderately high school refusal” profiles, which score high or moderately high on the four dimensions of SRAS-R. They also mention, although to a lesser degree, profiles such as that of “moderately low school refusal,” “school refusal due to tangible reinforcement,” “school refusal due to negative reinforcement,” and “school refusal due to positive reinforcement.”

There is limited research on the study of SR profiles that use samples of children equivalent to that used in this work. Four investigations have been found, conducted on Spanish students aged 8 to 12 (Gonzálvez et al., 2018, 2019, 2021; Martín et al., 2021). All of these studies have determined the existence of a profile called “non-SR or low SR” whose scores are low in all of the assessed dimensions. With the exception of one of them, which only addressed the study of dimensions I and IV of the SARS-R (Gonzálvez et al., 2019), the remainder referred to the existence of a “mixed profile,” maintained by distinct types of reinforcement. Finally, it should be noted that all research assumes the existence of an “SR by negative reinforcement” profile.

As occurred in studies using an adolescent sample, those involving children aged 8 to 12 have identified “mixed SR” as the most maladaptive profile, having worse results in almost all areas assessed on school anxiety (Gonzálvez et al., 2018), on social functioning (Gonzálvez et al., 2019), and on academic self-attributions (Gonzálvez et al., 2021). On the other hand, the “SR by negative reinforcement” profile has been found to be more maladaptive in anxiety due to aggression (Gonzálvez et al., 2018) and in pessimism and neuroticism indices (Martín et al., 2021).

### 1.2 School refusal and health

The World Health Organization (WHO) has highlighted the association between health and education, pointing out the relationship between the former and a reduction in school dropout rates and with an increase in success and academic performance, as well as future employment opportunities and productivity (World Health Organization and UNESCO, 2021).

The correspondence between health and school attendance problems has been the subject of numerous studies, although the objectives proposed in the research differ significantly depending on the socioeconomic reality in which they are framed. Thus, researchers focusing their work on developing territories mainly focus their topics on factors that can be classified as basic vital needs, while in developed countries this situation is overcome and they concentrate their studies on factors which, although still relevant to the student’s socio-personal well-being, do not represent such a pressing vital need (Martínez-Torres et al., 2024). In any case, study results have supported the common notion that the consequences of school refusal are not limited to the absence of the school context, but are also likely to occur outside of it, in areas such as health.

Prior to the *Adolescent Child Health and Illness Profile* (CHIP-AE, Starfield et al., 1995), the evaluation of self-perceived health was unavailable for young people between 11 and 17 years of age. Later, the *Child Report Form* (CHIP-CE/CRF, Riley et al., 2004) was published, directed at a population aged between 6 and 11, adapted and validated to Spanish (Rajmil et al., 2003; Estrada et al., 2012). This instrument generates a profile with 5 domains: Satisfaction, Well-being, Resilience, Risks, and Performance. No domain effectively describes a child’s health on its own, since health status should be measured through the interrelation of the total (Riley et al., 2004).

### 1.3 Study objectives and hypotheses

The main objective of this study was to examine the relationship between SR and self-perceived health variables, through the analysis of latent profiles. It also aimed to analyze the relationship between the identified profiles and the assessed self-perceived health variables. Based on past studies, the following hypotheses were formulated:

*Hypothesis* 1: Different profiles will be distinguished for SR maintained by negative reinforcement based on the functional model of school refusal behavior (Kearney, 2002). Low scores on the first two factors of the SARS-R scale will define the “no risk of SR” profile. Moderate scores on these factors will correspond to the “moderate risk of SR” profile. High scores on the factors will correspond to the “high risk of SR” profile.

*Hypothesis* 2: Of the different profiles found, it is expected that the “high risk of SR” profile will be the most maladaptive as it correlates negatively with the health dimensions of the CHIP-CE/CRF, according to which positive scores are associated with good health.

## 2 Materials and methods

### 2.1 Participants

In order to guarantee the representativeness of the sample, participants were recruited through random cluster sampling. One or two schools were chosen randomly in each geographical area (north, south, east, west, and center) of the Spanish province of Alicante. In the first random selection, all schools agreed to participate in the study. Therefore, a second random selection was not necessary given the full cooperation of all educational institutions. As a result, 14 private, subsidized, and public schools participated in this study. Four groups were randomly chosen from each school. A total of 936 children made up the initial sample. Of these participants, 104 (11.1%) were excluded because their parents or legal guardians did not provide their written informed consent to participate in the study and 95 (10.15%) were excluded because they did not correctly fill out the self-report measures (for example, giving two responses to an item). The final sample consisted of 737 students (60.9% boys and 39.1% girls) aged 8 to 10 (*M* = 8.76, *SD* = 0.74). Table 1 presents the frequency distribution by sex and age. The sample presented a uniform distribution given that no differences were detected between groups: χ^2^ = 5.23, *p* = 0.073.

**Table 1:** Distribution of the sample by gender and age.

	8 years	9 years	10 years	Total
Male	206 28.0%	160 21.7%	83 11.3%	449 60.9%
Female	111 15.1%	126 17.1%	51 6.9%	288 39.1%
Total	317 43.0%	286 38.8%	134 18.2%	737 100.0%

### 2.2 Instruments

#### 2.2.1 School refusal assessment scale-revised

The SRAS-R, directed at youth aged 8 to 17, aims to evaluate the various motivations for school refusal expressed by students. It consists of 24 items made up of four factors: I. Avoidance of school stimuli that cause a feeling of general negative affectivity (SRAS-R I. E.g., “How many times have you tried not to go to school because if you go you will feel sad or depressed?”); II. Escape from social and/or assessment situations in the school (SRAS-R II. E.g., “How many times have you tried not to go to school because you are embarrassed to be in front of others in the school?”); III. Search for the attention of significant others (SRAS-R III. E.g., “How many times do you think about your parents or family when you are in school?”); and IV. Search for tangible reinforcement outside of the school (SRAS-R IV. E.g., “How often do you reject going to school because you want to have fun outside of school?”). This is a 7-point Likert-like scale (0 = never, 6 = always). For this study, the Spanish version of the SRAS-R (School Refusal Evaluation Scale-Revised, Gonzálvez et al., 2016) was used, which maintains the factorial structure of the original, but contains 18 items. It was decided to study those factors (SARS-R I and SARS-R II) that are maintained by negative reinforcement. For this study, adequate reliability coefficients were attained for the two factors analyzed using the Cronbach alpha test.: α = 0.73 (SRAS-R I), α = 0.71 (SRAS-R II).

#### 2.2.2 Child health and illness profile-child edition/child report

**Figure 1:**
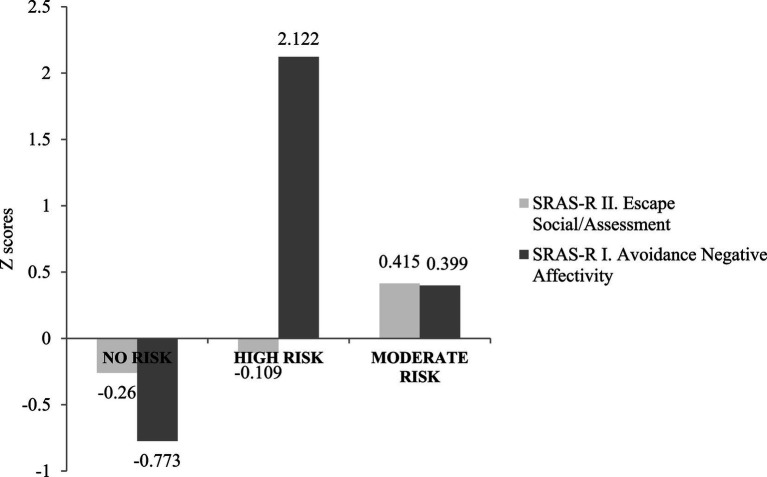
School refusal behavior profiles using Latent Profile Analysis (LPA).

The CHIP-CE/CRF is an instrument designed to evaluate perceived health in a sample of children aged 6 to 12. It consists of 45 items grouped into 5 health dimensions: I. Satisfaction, representing complacency with the child’s own health and self-esteem (e.g., “*How often do you feel very proud of yourself?*”); II. Well-being, which refers to physical and psychological symptoms and the limitation of activities (e. g., “*Over the past 4 weeks, how many times have you felt an intense pain in your stomach?*”); III. Resilience, which considers the states or behavior of the child that may improve their health in the future, in both an interpersonal manner, focusing on the support resources provided by the family, as well as in an intrapersonal manner, including activity items indicating physical aptitude (e. g., “*Over the past 4 weeks, how many times have you gotten along well with your parents?*”); IV. Risks, indicating behaviors and actions that may potentially act against the child’s health (e. g., “*Over the past 4 weeks that you have gone to school, how many times have you gotten into trouble in school?*”); V. Performance, which considers academic performance and the positive influence of peers (e. g., “*Over the past 4 weeks that you have gone to school, how has your reading gone?*”). The information is complemented with three sociodemographic questions (sex, age, and grade). A Likert-type scale is used, with 5 response options varying depending on the question asked (1 = never, 5 = always; 1 = no days, 5 = everyday; 1 = very bad, 5 = very well; 1 = bad, 5 = excellent) using circles that gradually increase in size depending on the answer (see Figure 1). At the same time, each item includes two figures at the edges of the response categories to facilitate understanding of the referred content. The highest scores indicate better health in all dimensions. For this study, adequate reliability coefficients were obtained using the Cronbach alpha test: α = 0.82 (Well-being), α = 0.77 (Satisfaction), α = 0.73 (Resistance), α = 0.70 (Risks), α = 0.74 (Functions).

### 2.3 Procedure

After obtaining approval from the Ethics Committee of the University of Alicante (UA-2022-09-29_2), for the study protocol, the necessary government permits were requested. Likewise, the purpose of the research was detailed to the school councils of each school and written informed consent was obtained from the parents and/or legal guardians of the students.

Subsequently, the participants collectively (groups of 15 to 25 students) completed the questionnaires in their classrooms during the school day. They were informed of the anonymous and voluntary nature of the tests. The average administration time was 35 min for both questionnaires. To standardize the administration process, a researcher explained the procedure, clarified possible issues, and verified the individuality of the responses. The ethical standards of the 1964 Declaration of Helsinki were followed at all times.

### 2.4 Statistical analyses

The analysis began by calculating the Pearson product–moment correlation coefficients, determining the gradation of the correlation itself through the use of Cohen (1988) classification, which proposed a small size for values equal to or greater than 0.10 and equal to or less than 0.29, a moderate size for those ranging from 0.30 to 0.49 and a high size for those exceeding 0.50.

The scores obtained in SRAS-R I and SRAS-R II were standardized, obtaining the corresponding z scores. These were interpreted by establishing limits of −0.5 and 0.5, with z scores lower than −0.5, suggesting low levels of school refusal, 0.5 suggesting high levels, and intermediate values between both null suggesting and moderate levels. Likewise, the Latent Profile Analysis (LPA) was used to determine the number of SR profiles based on negative reinforcement. To determine the possible number of sub-groups, the lowest values of the Bayesian Information Criteria (BIC) and the Akaike Information Criterion (AIC); the Vuong-Lo–Mendell–Rubin (LRT) likelihood ratio test, and the Bootstrap Likelihood Ratio Test (BLRT) were considered.

Once the profiles of school refusal based on negative reinforcement were known, a multivariate analysis of variance (MANOVA) was performed to determine the existence of a differentiated pattern between these profiles in the different dimensions of perceived health analyzed. To determine the existence of statistically significant differences, the Wilks Lambda value was used. To calculate the effect size, the Eta squared index was used (η2). *Post-hoc* tests (Scheffé’s method) were used, and the effect size was calculated using Cohen’s d (1988), distinguishing between a small (0.20 ≤ *d* ≤ 0.49), moderate (0.50 ≤ *d* ≤ 0.79), and large (*d* ≥ 0.80) size. For the calculations, the SPSS version 26 and MPlus version 8 were used.

## 3 Results

### 3.1 School refusal behavior profiles

Table 2 presents the relationship between the SRAS-R I (I. Avoidance of school stimuli that provoke negative affectivity) and SRAS-R II (II. Escape from social and/or assessment situations in the school context) factors and the five examined factors of the Spanish version of the CHIP-CE/CRF (I. Satisfaction, II. Well-being, III. Resilience, IV. Risks, and V. Performance).

**Table 2:** CRF Correlations between factors I and II of the SRAS-R and factors of the CHIP-CE/CRF.

CHIP-CE/CRF	SRAS-R I	SRAS-R II
Satisfaction	0.166^**^	−0.483^**^
Well-being	−0.462^**^	−0.224^**^
Resilience	n.s.	−0.143^**^
Performance	−0.112^**^	−0.429^**^
Risks	−0.119^**^	0.080^*^

**p < 0.001; *p < 0.05.

As for the SRAS-R I, significant correlations have been obtained in four of the factors outlined in the CHIP-CE/CRF (all with a *p* < 0.001), with the sole exception of the “Resilience” factor. Only one factor, “Satisfaction” (0.166, *p* < 0.001) has a positive correlation, while the others have presented manifest significance with negative correlations: “Well-being” (−0.462, *p* < 0.001), “Risks” (−0.199, *p* < 0.001), and “Performance” (−0.112, *p* < 0.001). Of all the correlations, only the one between “Well-being and SARS-R I” is moderate (almost high) in size, while the others are small in size.

Regarding SRAS-R II, significant correlations are found for the five factors established in the CHIP-CE/CRF. “Risks” (−0.080, *p* < 0.005) is the only factor having a positive correlation, small in size, while the other dimensions have negative correlations: moderate-sized “Satisfaction” (−0.483, *p*<0.001) and “Performance” (−0.429, *p* < 0.001); small-sized “Well-being” (−0.224, *p* < 0.001) and “Resilience” (−0.143, *p* < 0.001).

### 3.2 Latent profiles of school refusal maintained by negative reinforcement

In order to identify sets of participants based on their results on the SARS-R, various latent profile analyses were performed. Five models having two to six profiles were analyzed. Table 3 shows the resulting goodness-of-fit indices determined for the estimated models. All of the evaluated models have a *p* < 0.01 for the BLRT but only two, those with two and three profiles, had sizes equaling zero. This size was considered when determining the best model, assuming that the profiles had to make up at least 1% of the sample. Therefore, models with four, five, and six profiles were rejected, despite having lower AIC and BIC values and/or higher levels of entropy.

**Table 3:** Data fit of all models.

Models	AIC	BIC	BIC-adjusted	LRT *p*	LRT-adjusted	BLRT	Entropy	Size
2	3982.14	4014.36	3992.13	<0.001	<0.001	<0.001	0.904	0
3	3617.44	3663.46	3631.71	<0.001	<0.001	<0.001	0.956	0
4	3336.72	3396.55	3355.27	<0.001	<0.001	<0.001	0.959	1
5	3207.94	3281.58	3230.77	<0.001	<0.001	<0.001	0.965	1
6	3089.62	3177.07	3116.74	<0.001	<0.001	<0.001	0.934	2

Akaike Information Criterion (AIC) and the Bayesian Information Criteria (BIC); LRT, Vuong-Lo-Mendell–Rubin Likelihood-Ratio Test; BLRT, Bootstrap Likelihood Ratio Test.

Therefore, the latent profile model having the best fit of the data, and which was used to perform the following data analyses, was the one made up of three participant subgroups (3LC). Of the two models with size zero, this one obtained a higher entropy value (0.956) and lower AIC and BIC values (3617.44 and 3663.46 respectively). Like the rest, the *p* values for both LRT and BLRT were statistically significant (all with figures <0.001).

In the selected model, 3LC, the distribution of participant frequency and percentage was as follows: LC1 *n* = 377 (51.2%), LC2 *n* = 90 (12.2%), and LC3 *n* = 270 (36.6%). The first profile, LC1, consisted of students obtaining moderate-low scores on SR of the SRAS-R I type (z = −0.260) and significantly low scores in SRAS-R II (z = −0.773); This group is called “no risk of SR.” LC2 consisted of participants with low scores on SRAS-R I (z = −0.109) and significantly high scores on SRAS-R II (z = 2.122). This profile is defined as “high risk of SR.” The LC3 profile was available for boys and girls with moderate scores on both SRAS-R I (z = 0.415) and SRAS-R II (z = 0.399). Therefore, it was called “moderate risk of SR.” At all times, the SR was maintained by negative reinforcement (see Figure 1).

### 3.3 Relationship between latent profiles of school refusal on perceived health variables

Analysis was performed to determine if the students situated within each of the three latent profiles revealed specific and differential patterns with respect to the perceived health variables examined. The MANOVA highlighted statistically significant differences between the latent profiles for all of the perceived health dimensions (Wilks Lambda = 0.48, *F*_(10,734)_ = 65.44; *p* < 0.001, *η_p_^2^* = 0.31).

The “*no risk of SR*” profile obtained the highest averages on the dimensions of “*well-being”* and “*satisfaction,”* followed by “*performance*” and “*risks*,” with the lowest scores being those of “*resistance*” (see Table 4). Participants from the “*high risk of SR”* group obtained higher scores on “*risks*” and “*well-being,”* with lower scores on “*performance*,” “*satisfaction,”* and “*resilience*.” As for the “*moderate risk of SR*” profile, it had the highest averages on “*well-being,”* “*risks*,” and “*satisfaction,”* and lower averages on “*performance”* and “*resilience*.”

**Table 4:** Means and standard deviations obtained by the three clusters in self-perceived health dimensions.

	LC1		LC2		LC3		Statistical significance		
Dimensions	M	SD	M	SD	M	SD	F_(2,734)_	p	η^2^
Satisfaction	3.43	0.39	2.48	1.04	3.31	0.33	130.83	<0.001	0.26
Well-being	3.87	0.70	3.46	0.53	3.62	0.67	18.78	<0.001	0.05
Resilience	2.58	0.36	2.20	0.76	2.76	0.31	62.30	<0.001	0.15
Performance	3.29	0.35	2.54	0.78	2.95	0.52	99.01	<0.001	0.21
Risks	3.24	0.39	3.35	0.52	3.48	0.38	27.05	<0.001	0.07

LC1, “no risk of SR”; LC2, “high risk of SR”; LC3, “moderate risk of SR.”

*Post hoc* comparison studies found significant differences between the latent profiles considered and the perceived health dimensions assessed (see Table 5). Thus, in the comparison between the three profiles (LC1-LC2, LC1-LC3, and LC2-LC3), large effect sizes were found for the “*Satisfaction*” dimension, with the no risk profile (LC1) obtaining higher values, followed by the moderate risk profile (LC3), and finally, the high-risk profile (LC2). In the same comparison, the “*Resilience*” dimension had large or moderate sizes, with LC3 being the profile having the highest scores, followed by LC1 and LC2. The “*Performance*” dimension had a large effect size between LC1-LC2 and a moderate size between LC1-LC3 and LC2-LC3, revealing higher values on LC1 followed by LC3 and LC2. The “*Risks*” and “*Well-being*” dimensions revealed significant differences in some relationships but also showed moderate or low effect sizes. For “*Risks*,” LC3 had the highest scores and for “*Well-being*,” LC1 revealed the highest values.

**Table 5:** Cohen’s d value for *post-hoc* contrasts between cluster groups on self-perceived health dimensions.

Dimensions		LC1 vs. LC2	LC1 vs. LC3	LC2 vs. LC3
Satisfaction	*p*	<0.001	0.009	<0.001
	*d*	1.65	1.87	−1.40
Well-being	*p*	<0.001	<0.001	n. s.
	*d*	0.61	0.36	---
Resilience	*p*	<0.001	<0.001	<0.001
	*d*	0.82	−0.53	−1.21
Performance	*p*	<0.001	<0.001	<0.001
	*d*	1.62	0.79	−0.69
Risks	*p*	n. s.	<0.001	0.022
	*d*	---	−0.62	−0.31

LC1, “no risk of SR”; LC2, “high risk of SR”; LC3, “moderate risk of SR.”

Therefore, it has been confirmed that LC1, as compared to LC2 and LC3, had significantly higher values on “*satisfaction*” and “*performance*” (with a large effect size in both cases) and on “*well-being*” (with a moderate-low effect size on both). LC3 had higher values on “*resilience*” (with a large effect size as compared to LC2 and a moderate one as compared to LC1) and “*risks*” (with a moderate or low effect size). LC2 had lower scores compared to LC1 and LC3 on “*satisfaction*,” “*resilience*,” and “*performance*” (with a large or moderate effect size, depending on the comparison).

## 4 Discussion

The objective of this study was to examine the relationship between SR and the self-perceived health variable in a community sample of students aged 8 to 10. Various working hypotheses were established which have been demonstrated according to the results obtained when applying the SRAS-R scale to assess refusal behavior, and the CHIP-CE/CRF questionnaire to analyze self-perceived health. This study focused on the analysis of SR behavior maintained by negative reinforcement, leading the way for more specific future interventions on individual students, given that this student profile presents some of the most maladaptive values.

Regarding the first hypothesis, referring to the existence of distinct profiles of students with SR maintained by negative reinforcement, three profiles have been confirmed: a “*no risk*” of SR profile (51.2% of the students), in line with the findings from prior studies; a “*moderate risk*” profile, presenting moderate scores on both the SRAS-R I and the SRAS-R II factors (36.6% of the students); and a “*high risk*” profile presenting high scores on the SRAS-R II factor (12.2% of the students). These findings reveal a student body that requires action, given its high risk of presenting SR behaviors, but worryingly, the results also suggest that many students are at moderate risk of exhibiting such behaviors, students whose profile could increase to *high-risk* if their needs are not met.

In response to the second hypothesis, the scientific literature has identified the profiles of “mixed SR” and “SR by negative reinforcement” as the most maladaptive for certain factors, including when considering positive reinforcement maintaining SR (Gonzálvez et al., 2018, 2019, 2021; Martín et al., 2021). In this study, it has been confirmed that having a greater risk of SR maintained by negative reinforcement is associated with factors that affect the adaptive behavior of students. Thus, the profile that is not at risk of SR obtains significantly higher scores, with moderate or high effect sizes as compared to the moderate or high risk profiles, in the areas of “satisfaction,” “well-being,” and “performance.” The moderate-risk profile only scores higher on the “resilience” factor, as compared to the other two profiles. The “high risk” profile, which is determined to be the most maladaptive of the three, receives the lowest scores in all areas except for the “risks” factor (which does not present significant effect sizes for the comparison between any of the profiles).

Considerable research has been conducted on SR behavior and its association with numerous variables. Specifically, in the area of health, it has been found that non-specific somatic symptoms may be related to school refusal behavior (Li et al., 2021) and students displaying school absenteeism behavior have a lower quality of life in terms of health, as compared to the control group (Van Den Toren et al., 2019; Matsuura et al., 2020). However, additional research on the relationship between health and SR behavior is necessary. Therefore, the results of this study help to fill the existing gap, while encouraging individualization in educational interventions and the addressing of certain school attendance problems through more specific future actions.

The scope of this work covers the gap existing in research on SR behavior maintained by negative reinforcement since no previous works have addressed the association between SRAS-R I and SRAS-R II dimensions and health variables. Thus, without overlooking that no factor should be considered in isolation (Riley et al., 2004), it is confirmed that, overall, the presence of SR maintained by negative reinforcement correlates negatively with perceived health dimensions. The assessed health dimensions have a negative correlation for SRAS-R I, except for “*resilience*” (which does not have a significant relationship) and “*satisfaction*,” whose correlation is positive and small in size. As for SRAS-R II, all of the dimensions have a negative relationship except for “*risks*” (with a small size).

## 5 Limitations and future perspectives

Despite the novelty of its objectives, this study suffers from certain limitations. A contrast appears to exist between the students’ self-perceived health scores and scores from other instruments that have not been used in this study. Furthermore, it is unknown whether the determined patterns of both health and school refusal will remain stable over time, with longitudinal studies being necessary to determine this. Future research may examine SR profiles maintained by positive reinforcement and mixed profiles, their relationship with health, and their ability to adapt to the environment.

In conclusion, the analysis of latent profiles has confirmed the existence of distinct profiles of students with SR that are maintained by negative reinforcement. It has also been found that this type of school refusal correlates negatively with health dimensions, whereby a greater presence of SR predicts worse self-perceived health in students aged 8 to 10. It has also confirmed that the profile having the highest risk of expressing school refusal due to negative reinforcement is also the most maladaptive. These findings promote relevant clinical implications by identifying students who need intervention, not only because they are at high risk of exhibiting SR behaviors but also because they have moderate scores whose risk profile could change if their needs are not met. These implications will be focused on the individualization of both the socio-educational intervention and the approach of specific preventive actions on students who are at high risk.

## Data availability statement

The raw data supporting the conclusions of this article will be made available by the authors, without undue reservation.

## Ethics statement

The studies involving humans were approved by Ethics Committee of the University of Alicante. The studies were conducted in accordance with the local legislation and institutional requirements. Written informed consent for participation in this study was provided by the participants’ legal guardians/next of kin.

## Author contributions

JM-T: Writing – original draft. CG: Writing – review & editing. NA: Writing – review & editing.
